# *Burkholderia gladioli* strain KJ-34 exhibits broad-spectrum antifungal activity

**DOI:** 10.3389/fpls.2023.1097044

**Published:** 2023-03-03

**Authors:** Chunnan Yang, Zhihui Wang, Jiangxue Wan, Tuo Qi, Lijuan Zou

**Affiliations:** ^1^Ecological Security and Protection Key Laboratory of Sichuan Province, Mianyang Normal University, Mianyang, China; ^2^Kaijiang County Plant Protection and Quarantine Station, Kaijiang County Agricultural and Rural Bureau, Dazhou, Sichuan, China; ^3^State Key Laboratory of Crop Gene Exploration and Utilization in Southwest China, Sichuan Agricultural University at Wenjiang, Chengdu, Sichuan, China

**Keywords:** biocontrol, *Burkholderia gladioli*, antifungal, fungal pathogens, *Botrytis cinerea*, metabonomics

## Abstract

**Introduction:**

Plant pathogens are one of the major constraints on worldwide food production. The antibiotic properties of microbes identified as effective in managing plant pathogens are well documented.

**Methods:**

Here, we used antagonism experiments and untargeted metabolomics to isolate the potentially antifungal molecules produced by KJ-34.

**Results:**

KJ-34 is a potential biocontrol bacterium isolated from the rhizosphere soil of rice and can fight multiple fungal pathogens (i.e. *Ustilaginoidea virens, Alternaria solani, Fusarium oxysporum, Phytophthora capsica, Corynespora cassiicola*). The favoured fermentation conditions are determined and the fermentation broth treatment can significantly inhibit the infection of *Magnaporthe oryzae* and *Botryis cinerea*. The fermentation broth suppression ratio is 75% and 82%, respectively. Fermentation broth treatment disrupted the spore germination and led to malformation of hyphae. Additionally, we found that the molecular weight of antifungal products were less than 1000 Da through semipermeable membranes on solid medium assay. To search the potentially antifungal molecules that produce by KJ-34, we used comparative and bioinformatics analyses of fermentation broth before and after optimization by mass spectrometry. Untargeted metabolomics analyses are presumed to have a library of antifungal agents including benzoylstaurosporine, morellin and scopolamine.

**Discussion:**

These results suggest that KJ-34 produced various biological control agents to suppress multiple phytopathogenic fungi and showed a strong potential in the ecological technologies of prevention and protection.

## 1 Introduction

Plant-pathogenic fungi cause yield losses and decreased quality of cereals and vegetables, thereby resulting in enormous economic losses and threats to food security worldwide (Wang et al., 2022). Resistance gene exploration and utilization, optimum cultivation measures, and appropriate amounts of chemical fertilizers and pesticides are considered to be the main strategies in plant protection (Peng et al., 2021; Kumar et al., 2022). However, the control of phytopathogens by commercial fungicides often leads to the development of fungicide resistance, and the Food and Agricultural Organization [FAO (www.fao.org)], in November 2021, issued the United Nations Food and Agriculture Organization Action Plan on Antimicrobial Resistance (2021–2025) to combat the serious global threat posed by antimicrobial resistance (Umair et al., 2021). Modern biological control requires a comprehensive understanding of the antagonistic and growth-promoting mechanisms of microorganisms, by clarifying the relationship among biocontrol factors, the environment, and pathogens in the ecosystem. It is then necessary to adjust the dynamic balance between exogenous biocontrol factors and pathogens in the implementation of biological control measures, so as to achieve the optimum balance between supply of and demand for the use of biocontrol agents in the process of disease control and to meet the requirement of not destroying the ecological environment. It is of great importance, for the prevention and control of fungal diseases, to explore new microorganisms with bionic potential and to analyze their bionic mechanisms (Badescu et al., 2022).

Soil is composed of mineral particles, organic matter, and microorganisms; the co­evolution of microorganisms and their effects on fungal plant pathogens remain unclear (Joo and Hussein, 2022). However, the antimicrobial activity of antagonistic microbes (i.e., *Bacillus*, *Pseudomonas*, *Agrobacterium*, and *Actinomycetes*) and their natural products show great potential for use in sustainable strategies of plant protection and green pesticides development (Chakraborty et al., 2021; Han et al., 2022). The genus *Burkholderia*, which is widespread in natural environments with host diversity (Compant et al., 2022) produces bongkrek acid and toxoflavin, which can cause food poisoning (Elshafie and Camele, 2021). Burkholderia glumae and B. gladioli (i.e., B. gladioli BSR3, B. gladioli RSB1, and B.gladioli RSB15), known to be plant pathogens, can cause rice sheath (i.e., BSR3, RSB1, RSB10, and RSB15) and can also infect other plant species (*Gladiolus*, onions, and mushrooms) (Lee et al., 2021; Chaeyeong et al., 2018). However, several other *Burkholderia* species play a positive role in protecting plant offspring against phytopathogens by producing a wide variety of antimicrobial compounds (e.g., reumycin, sinapigladioside, protein, oligopeptide, pyrrolnitrin, and bactobolins) (Gu et al., 2009; Schmidt et al., 2009; Dose et al., 2021; Swain et al., 2017). The genus *Burkholderia*’s own antimicrobial activity produces data that are useful for agricultural drug discovery, and which are important for sustainable aquaculture (Depoorter et al., 2021).

Owing to the great potential of *Burkholderia* bacteria in biocontrol, we isolated the *B. gladioli* strain KJ-34, which has antifungal properties; we also tested the resistance spectrum of plant-pathogenic fungi and found a broad-spectrum resistance to various filamentous fungi. The biological control agents produced by KJ-34 suppressed spore germination and hyphal growth in *M. oryzae* and *B. scinerea*. We also analyzed the biological control metabolites using metabolomics. These discoveries show great promise for sustainable agriculture and new fungicide development.

## 2 Materials and methods

### 2.1 Isolation and identification of biocontrol bacterium

Rhizosphere soils were collected from Kaijiang, Sichuan province (31°08′N, 107°87′E). Rhizosphere soils were diluted with sterile water and dilutions of 10^–3^ to 10^–9^ were selected for bacterial isolation. Bacteria were separated on nutrient agar (NA) and potato dextrose agar (PDA) at 28°C. All morphologically distinct colonies were collected for confrontation culture on PDA with the indicator strains *M. oryzae* and *B. cinerea*. Moreover, 16S was amplified by the specific primer (27F: AGA GTT TGA TCM TGG CTC AG; 1,492R: CGG TTA CCT TGT TAC GAC TT) (Lane, 1985) and the phylogenies were reconstructed using the neighbor-joining (NJ) method using MEGA 7.0 software (Kumar et al., 2016).

### 2.2 Antagonism experiments against plant pathogenic fungi

The antifungal activity of 183 diverse bacterial species was tested in a crossing-culture assay against *B. cinerea* and *M. oryzae*: 18 species showed a clear zone of growth inhibition. For further antagonistic tests, KJ-34 was inoculated on one side of a PDA plate and the fungi (*Ustilaginoidea virens*, *Alternaria solani*, *Fusarium oxysporum*, *Phytophthora capsica*, *Corynespora cassiicola*, *Magnaporthe oryzae*, *Rhizoctonia solani*, *Bipolaris maydis*, *Botrytis cinerea*, *Colletotrichum lagenarium*, *Epicoccum nigrum*, *Fusarium proliferatum*, *Alternaria brassicae*, and *Phomopsis vexans*, which are maintained by the Laboratory of Integrated Plant Disease Control, Northwest Agriculture and Forestry University) mycelial plugs were inoculated on the other side after KJ-34 growth at 28°C for 1 day. At least three plates were used for each phytopathogenic fungus.

### 2.3 Optimization of culture conditions and shaking flask fermentation process

The culture media used in this study included Luria-Bertani (LB), Potato Dextrose Agar (PDA), Potato-Saccharose-Agar (PSA), Nutrient Agar (NA), Complete medium (CM), Ashby nitrogen-free medium and Tryptic Soytone Agar (TSA), Ashby nitrogen-free medium, and TSA. The optimum culture medium for measuring the antifungal activity of KJ-34 was determined by testing various combinations of different carbon components (sucrose, fructose, maltose, xylose, galactose, raffinose, mannose, sorbitol, corn flour, etc.) and nitrogen components (NH_4_Cl, (NH_4_)_2_SO_4_, KNO_3_, NH_4_NO_3_, beef paste, peptone, yeast extract, potato powder, etc.) Different culture conditions, such as fermentation time, fermentation temperature, and shaker rotation speed, were also tested to determine the optimum conditions

### 2.4 Pathogen infection experiments

The *M. oryzae* strain Guy11 was incubated on CM at 28°C for 5 days. For *M. oryzae*, the spores were collected and sprayed on 14-day-old rice leaves (LTH) with a final concentration of 1 ×10^5^ (Sakulkoo et al., 2018). Lesions produced in each treatment were counted. *B. cinerea* was obtained from a local greenhouse and was incubated on PDA medium at 28°C for 5 days. The clumps of *B. cinerea* were placed on the center of the leaves of tomato landraces, and lesion areas were photographed and measured using ImageJ. Spores and mycelia were observed under an optical microscope (Olympus, Japan).

### 2.5 KJ-34 specialized metabolites identified by untargeted metabolomics

For the identification of specialized metabolites produced by KJ-34, KJ-34 was shaken (5 days, 28°C, 200 rpm) in PDB medium under optimum flask culture conditions; samples were collected and stored at − 80°C prior to ultra-performance liquid chromatography (UPLC)–tandem mass spectrometry (MS/MS) analysis using equipment manufactured by Metware Biotechnology Co., Ltd ., Wuhan, China.

## 3 Results

### 3.1 Isolation and identification of KJ-34

**Figure 1:**
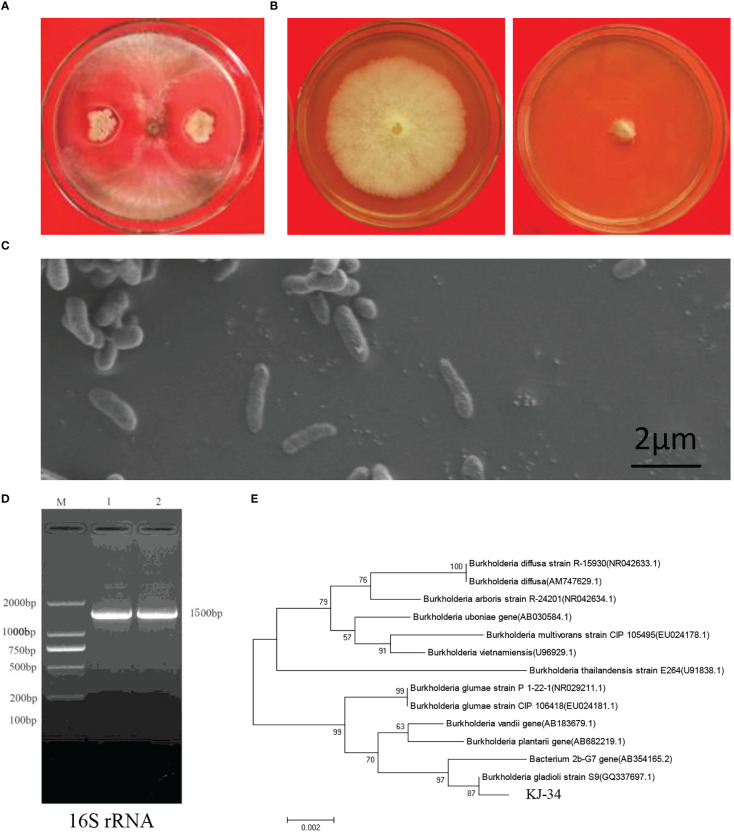
Identification of KJ-34. **(A)** Antagonistic inoculation of KJ-34 was applied to B. cinerea colonies grown on plates containing the solid medium PDA, and incubated at 28°C for 5 days, following which the colonies were photographed. **(B)** Inhibition of B. cinerea following treatment with KJ-34 shaking flask fermentation filtrate. **(C)** The bacterium was observed with a scanning electronic microscope (bar =2 μm). **(D)** 16S rRNA from KJ-34 was amplified by using the 27F/1,492 primer. **(E)** Theh phylogenetic tree of strain KJ-34 and its close relatives based on 16S rRNA gene sequencing. Dendrograms were generated by the neighbor-joining method.

Antagonism between microorganisms has attained a broad consensus: biocontrol strains from local areas of agricultural farms are eco-friendly and important for sustainable agriculture. To screen the antifungal activities of bacteria, rhizosphere soils were collected from local rice paddies in Kaijiang, Sichuan province (31°08′N, 107°87′E). Bacteria were separated on NA and PDA at 28°C. A total of 183 morphologically distinct bacterial species were tested for their antifungal activity in crossing-culture assay against *B. cinerea* and *M. oryzae*: 18 of them showed a clear zone of growth inhibition (**Figure S1**). Among these strains, KJ-34 exhibited the highest antifungal activity on the PDA plate; we then confirmed its antagonistic activities against *B. cinerea*, as shown in **Figures 1A, B**. Strain morphology was also observed using a scanning electron microscope (**Figure 1C**): 16S rRNA was amplified and sequenced to construct the phylogenetic tree, which revealed a high degree of similarity with *B. gladioli* (**Figures 1D, E**).

### 3.2 Growth profiles of strain KJ-34

In order to determine the most efficient culture medium for the isolation of antifungal compounds, we tested the growth velocity of KJ-34 on different culture media (LB, PDA, PSA, NA, and TSA) by measuring the bacterial concentration at 6, 12, 24, 48, 72, 96, and 120 hours. As shown in **Figures S2A, B**, in the first 48 hours of shaking flask fermentation, KJ-34 grew most rapidly in TSB, whereas after 72 hours, growth of KJ-34 was most rapid on PDB medium and the OD600 reached 1.94 (TSB and PDB are fluid medium of TSA and PDA without agar). The morphology of KJ-34 grown on solid medium was markedly different from that of KJ-34 subjected to shaking flask fermentation, although the growth ratio was similar. As shown in **Table 1**, the optimum flask culture conditions were selected through orthogonal tests; the optimum flask culture conditions were 2% cornmeal, 1.5% beef extract, and 0.01% FeSO_4_ (5 days, 28°C, 200 rpm). For the antifungal activity against *M. oryzae*. As shown in **Figure S3**, the antifungal activity of KJ-34 against M. oryzae was maximized by using CM as the fermentation broth and by shaking flask fermentation at 28°C in PDB for 6 days; the antifungal activity against *M. oryzae* was significantly increased by shaking for 5 days. Growth of To test the inhibition of KJ-34 shake flask fermentation against plant pathogen (6 days, 28°C, 200 rpm).

**Table 1:** Culture conditions and shaking flask fermentation optimized.

	Category	Diameter (mm) of inhibition zones
Carbon sources	Cornmeal	31.20 ± 0.62
	Galactose	30.27 ± 2.34
	Mannitol	27.93 ± l.50
	Glucose	23.53 ± 0.67
	Xylose	15.37 ± 0.40
	Glycerin	25.33 ± 0.45
	Fructose	23.10 ± 0.87
Nitrogen sources	Beef extract	33.50 ± l.40
	Pepton	33.23 ± 1.34
	Soybean powder	33.17 ± 0.98
	NH_4_Cl	32.53 ± 0.67
	NH_4_NO_3_	27.37 ± 1.9
	(NH_4_)_2_SO_4_	28.37 ± 0.42
Trace elements	0.01% FeSO_4_	34.53 ± l.19
	0.1% CaCO_3_	34.00 ± l.30
	0.1% KH_2_PO_4_	31.2 ± 1.08
	0.1% MgSO_4_	31.20 ± 1.08
	1% NaCl	30.23 ± 0.90
Orthogonal tests	1	33.13 ± 0.85
	2	34.37 ± 0.95
	3	36.03 ± 0.68
	4	35.10 ± l.15
	5	36.77 ± l.12
	**6**	**37.27 ± 0.85**
	7	33.57 ± l.46
	8	33.67 ± 0.67
	9	36.83 ± l.86

Bold values indicate optimum flask culture conditions.

### 3.3 Effects of KJ-34 metabolites on tomato gray mold disease

**Figure 2:**
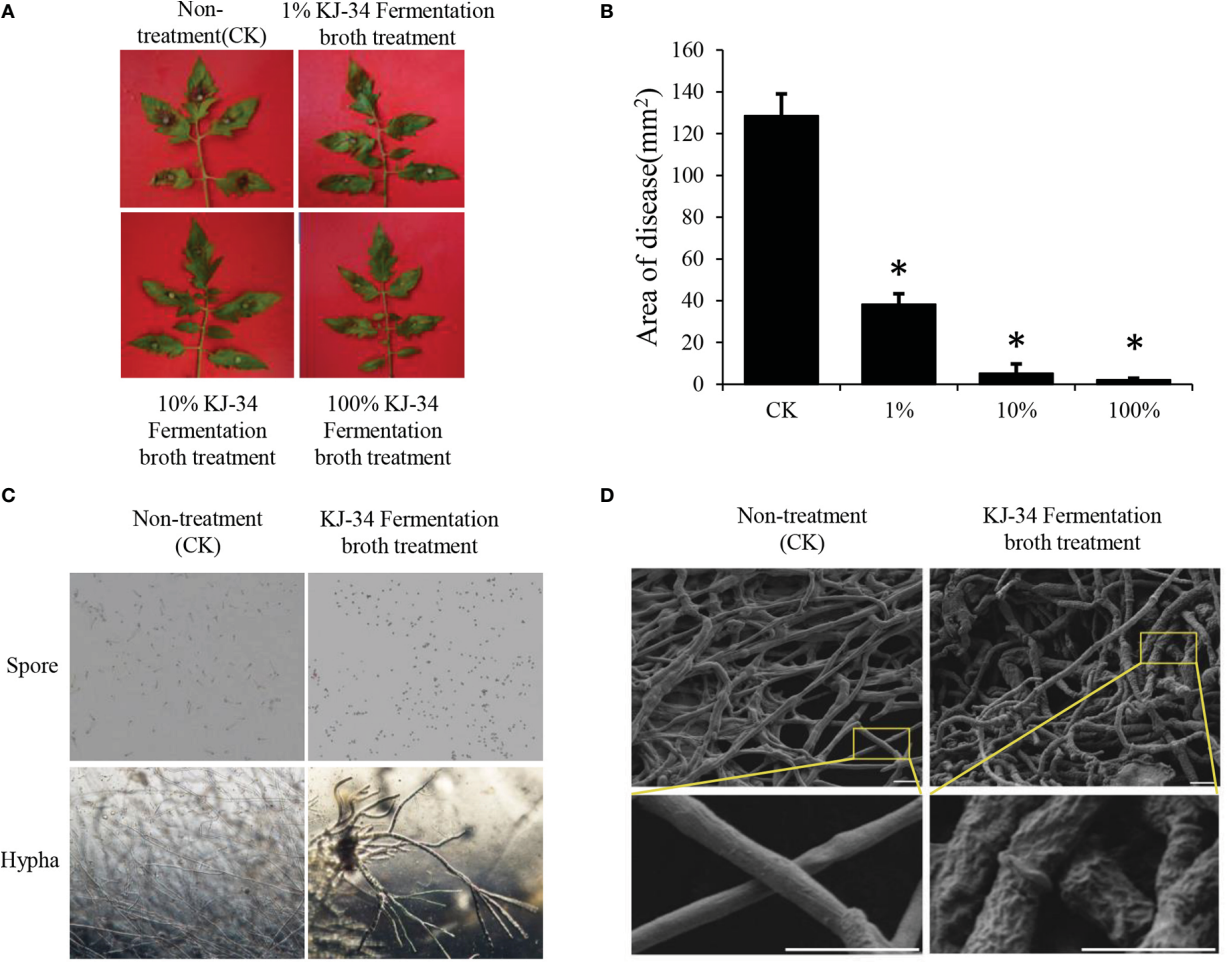
Biological control effect of KJ-34 fermentation filtrate against *B. cinerea*. **(A)** Tomato leaves were sprayed with the KJ-34 fermentation filtrate before inoculation with *B*. *cinerea*. Tomato leaves were treated with PDB as a control 5 days prior to pathogen inoculation. **(B)** Quantification of the lesion area of leaves pretreated with different concentrations of KJ-34 fermentation filtrate. Error bars represent the standard error of mean (*n* = 18). Asterisks indicate a significant difference (*p* < 0.05) according to the least significant difference test. **(C)** The effect of KJ-34 fermentation filtrate on spore germination and hyphal growth of *B*. *cinerea*. **(D)** The changes in mycelium morphology after KJ-34 fermentation filtrate treatment were observed with a scanning electronic microscope (bar = 2 μm).

To test the inhibition of KJ-34 shake flask fermentation against plant pathogen, we used *B. cinerea* as the representative strain. After 5 days of fermentation under optimum flask culture conditions, the fermentation broth was filtered and the filtrate diluted (concentrations: 100%, 10%, and 1%). As shown in **Figure 2A, B** and **Table S1**, the disease inhibition rate was 98.2% after treatment with 100% fermentation broth had a control efficiency of 75.6%, but the 1% fermentation broth treatment was also quite effective, with a control efficiency of 45.8%. Furthermore, the protective effect of KJ-34 fermentation filtrate was also analyzed, as shown in **Table 2**: control efficiency of complete fermentation broth treatment was 75.6%, and even the centesimal fermentation broth treatment was 45.8%. KJ-34 is thought to have a great developing potential in the control of plant fungicidal disease. To understand the effects of the KJ-34 strain on the growth of *B. cinerea*, the bacterial broth-treated spore germination was observed, as shown in **Figure 2C, D**. Spore germination in B. cinerea was inhibited by 24 hours’ treatment with KJ-34 metabolites and the results are consistent with the effective inhibition of tomato gray mold disease. The *B. cinerea* hypha were also swollen and deformed after co-culture. Scanning electron microscopy of mycelia also showed the same results. These results indicate that KJ-34 produces various antifungal metabolites, for use in the development of bactericides and fungicides.

**Table 2:** Inhibition spectrum of JK-34 fermentation filtrate.

Target pathogen	Inhibitory zone diameter (mm)
*Magnaporthe oryzae*	35.3 ± 2.5
*Ustilaginoidea virens*	15.7 ± 3.8
*Rhizoctonia solani*	11.6 ± 2.3
*Bipolaris maydis*	33.3 ± 6.7
*Botrytis cinerea*	35.4 ± 5.3
*Colletotrichum lagenarium*	41.2 ± 1.3
*Alternaria solani*	37.1 ± 1.2
*Fusarium oxysporum*	35.1 ± 0.9
*Phytophthora capsici*	32.3 ± 1.1
*Epicoccum nigrum*	31.2 ± 0.9
*Fusarium proliferatum*	26.2 ± 0.7
*Alternaria brassicae*	25.2 ± 0.7
*Phomopsis vexans*	25.1 ± 1.6
*Corynespora cassiicola*	22.5 ± 1.1

### 3.4 Effects of KJ-34 metabolites on blast disease

**Figure 3:**
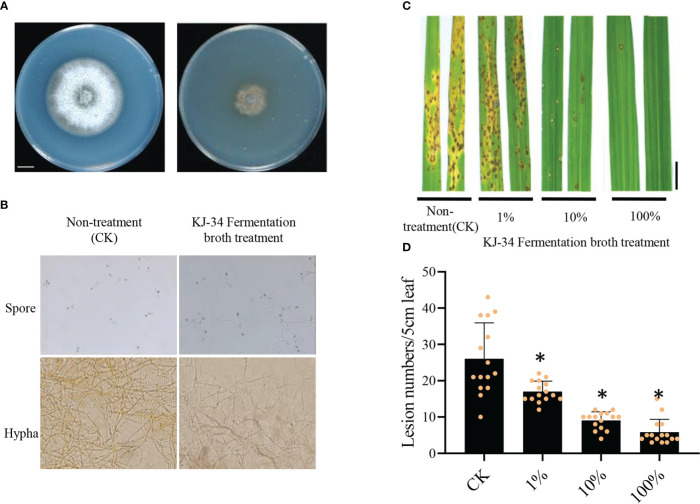
Biological control effect of the KJ-34 fermentation filtrate against rice blast. **(A)** Effects of the KJ-34 fermentation filtrate on the growth of **(B)** *M. oryzae*. *M. oryzae* was photographed after being inoculated for 6 days. **(C)** Rice leaves sprayed with the KJ-34 fermentation filtrate before inoculation with Guy11 spores. **(D)** Blast disease lesion density was quantified from infected leaf segments of 5 cm in length at 5 days post infection (*n* = 15 independent leaves; mean ± SD, two-sided Student’s *t*-test). *p < 0.05 (two-sided t-test).

To test the ability of the KJ-34 shaking flask fermentation filtrate to inhibit rice blast fungus, the fermentation broth was filtered after 5 days of fermentation with the optimum flask culture conditions added in CM medium. As shown in **Figure 3A**, the growth of M. oryzae were significantly inhibited and the bacterial broth treated spore germination and mycelial growth were observed, as shown in **Figure 3B**, M. oryzae spore produced more appressorium thus was failure to complete infection. The germinal tubes were also malformed. The *M. oryzae* hypha were swollen and deformed by co-culture with KJ-34 on plates containing solid PDA medium. Moreover, the fermentation broth was filtered after 5 days of fermentation under optimum flask culture conditions and the fermentation filtrate was then diluted (concentrations: 100%, 10%, and 1%). Spores of *M. oryzae* were mixed with the diluted fermentation filtrate and sprayed on to the rice leaves (**Figure 3C, D**): lesion numbers reduced by 34.6% after treatment with 1% fermentation filtrate, and by 77.5% after treatment with 100% fermentation filtrate. The results are consistent with the effective inhibition of tomato gray mold disease. These results indicate that KJ-34 has great potential in plant protection.

### 3.5 KJ-34 has broad-spectrum antifungal activity

**Figure 4:**
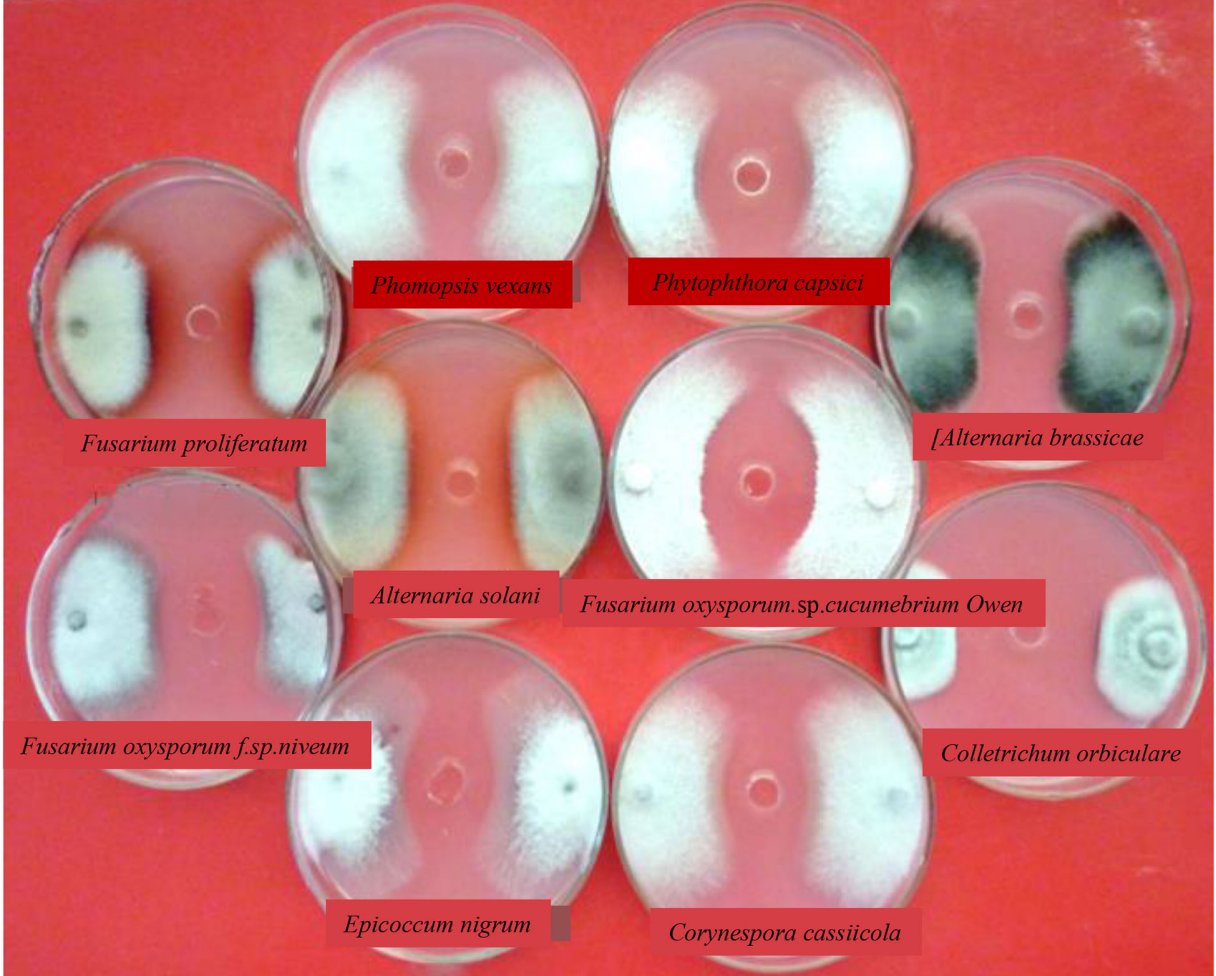
The antimicrobial spectrum of KJ-34 against plant pathogenic fungi.

Fungal growth inhibition was observed in the co-culture experiments. As shown in **Figure 4** and **Table 2**, several well-known fungi (*Ustilaginoidea virens*, *Alternaria solani*, *Fusarium oxysporum*, *Phytophthora capsica*, *Corynespora cassiicola*, *Magnaporthe oryzae*, *Rhizoctonia solani*, *Bipolaris maydis*, *Botrytis cinerea*, *Colletotrichum lagenarium*, *Epicoccum nigrum*, *Fusarium proliferatum, Alternaria brassicae*, and *Phomopsis vexans*) that are pathogenic to crops, vegetables, or fruits were grown along with KJ-34 on solid PDA medium. KJ-34 strongly inhibited the filamentous growth of the fungal pathogens tested. KJ-34 co-culture with *C. lagenarium* significantly inhibited growth of the fungus, and the diameter of the inhibitory zone was 41.2 mm. The growth of other fungi co-cultured with KJ-34 was also inhibited, but the magnitude of the inhibitory effect did not depend on the concentration of the fermentation filtrate. The flat growth restraint of the other fungi co-cultures with KJ-34 also showed an obviously inhibitory effect. The rice-pathogenic fungi *U. virens* and *R. solani* were only weakly inhibited, largely because their growth rate was either too fast or too slow, and another reason could be because of the nutrient competition and the accompanying of antifungal compounds change.

### 3.6 Exploring the antifungal activity compounds of KJ-34

**Figure 5:**
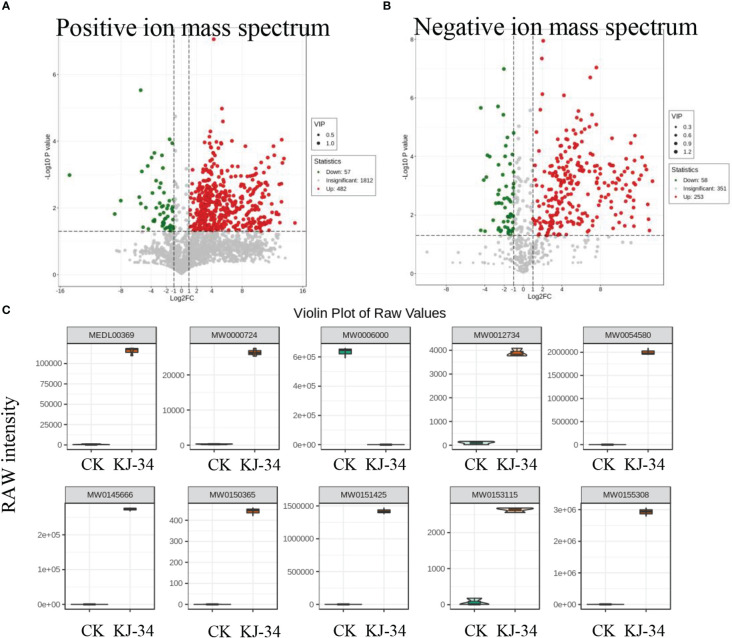
Specialized metabolites produced by KJ-34. **(A, B)** Volcanic map of differential metabolites. Each dot in the volcano plot represents a metabolite, with green dots representing differentially down-regulated metabolites’ and differentially upregulated metabolites, red dots up-regulated differential metabolites, and gray metabolites detected but not significantly different. **(C)** Violin plot of some differential metabolites significantly enriched in KJ-34 fermentation broth.

KJ-34 appeared to produce antimicrobial metabolites effective against multiple phytopathogenic fungi. To explore the antifungal activity compounds produced by KJ-34, we first dialysis the medium optimized with 10 kDa filter attenuates and then 1 kDa (Li et al., 2019). The metabolites with a molecular weight below 1,000 Da produced by KJ-34 also exhibited high inhibitory ability and a broad antibiotic spectrum (data not shown). With these results, we performed the untargeted metabolomics to explore the pronounced inhibitory effects of KJ-34 against *M. oryzae* as compared with the medium before optimized. As shown in **Figure S4** and **Table S2 and S3**, 2,352 metabolites were included in the positive ionization model and 663 metabolites were included in the negative ionization model. The detected metabolites were subjected to qualitative and quantitative analyses and, based on the results, divided into groups. Fold changes in the quantitative information for the metabolites in each group were then compared. Differential metabolites with a fold change of ≥ 6 are listed in **Tables S3, S4**, and metabolites annotated in accordance with the KEGG (Kyoto Encyclopedia of Genes and Genomes) pathway database are displayed in **Figure S4**. Differential metabolites are involved in 2-oxocarboxylic acid metabolism, ovarian steroidogenesis, protein digestion and absorption, serotonergic synapse pathway, steroid hormone biosynthesis, bile secretion, porphyrin and chlorophyll metabolism, and arachidonic acid metabolism. After optimizing the flask culture conditions, metabolites significantly increased in both type and quantity (**Figure 5A, B**, **Figure S4**).

We also analyzed the differential metabolites from multiple perspectives according to the characteristics of publicly available databases, and finally accurately analyzed the main differential antifungal compounds that may be responsible for the broad antibiotic spectrum (**Figure 5C**). Metabolites marked with a yellow background in **Table S3 and S4** (Ac-Yvad-cho, benzoylstaurosporine, TAXOL C, morellin, jubanine B, trichostatin A, thapsigargin, kabiramide B, scopolamine, enniatin B, latrunculin A, rifaximin, rigin, and garcinone C) appear to be promising bioactive compounds. These results are consistent with antifungal activity against a wide range of phytopathogenic fungi. Moreover, oligopeptides were identified in the metabolome; whether or not the oligopeptides are functional needs further validation (**Tables S4 and S5**). KJ-34 is not surprising to be a library of antifungal compounds.

## 4 Discussion

Sustainable agriculture is a new goal of modern agriculture, with the aim of achieving higher yields while at the same time ensuring food security (Verma et al., 2022; Kumari et al., 2022). Biotic stresses, especially pathogenic fungi, greatly threaten global food security but chemical fungicides are indiscriminate and often overused. Biocontrol is a traditional, effective, and sustainable solution for food security. The discovery of new microorganisms that can be used as biocontrol agents is attracting researchers’ attention (Pradhan et al., 2022). In this study, we isolated and characterized biocontrol strain KJ-34, which exhibited high antifungal activity against a broad range of fungal phytopathogens. By optimizing fermentation conditions, KJ-34 was able to produce a greater variety of antifungal compounds, and in greater quantities. These compounds were found to suppress spore germination and mycelial growth in *M. oryzae* and *B. cinerea*. KJ-34 have protective and therapeutic effects for the fungal disease. The metabolites produced by KJ-34, which may be responsible for KJ-34’s broad antibiotic were preliminary identified by comparative and bioinformatic analyses of metabolome. KJ-34 has great potential for use in the development of antibiological inoculants and eco-friendly fungicides.

*Burkholderia* bacteria are widespread, have versatile metabolism and have a range of host species, which include pathogenic and plant probiotics members (Adaikpoh et al., 2022). *Burkholderia gladioli* BSR3 infects rice, causing bacterial blight, and another rice seed−borne bacterium, *B. glumae*, causes rice grain rot (Jeong et al., 2003; Seo et al., 2011). On the other hand, many *Burkholderia* bacteria have the ability to promote plant growth and to act as biofertilizer, such as the *B. vietnamiensis* strain TVV74, which produced a 22% increase in rice yield as a result of its nitrogen fixation activity (Van et al., 2000; Parke and Gurian-Sherman, 2001). Some strains of *Burkholderia* bacteria occupy dominant ecological niches in the endophytic bacterial community, to prevent the reproduction of pathogens (Hallmann et al., 1999; Ajiboye et al., 2022). Some seed-borne *Burkholderia* bacteria promote growth by producing plant hormones (Kloepper, 1993).

KJ-34 also produces various hormones and hormone-related compounds and its ability to promote plant growth is worth exploring in the future. KJ-34 was also found to fix nitrogen *in vitro* when grown on a nitrogen-deficient medium (Ashby nitrogen-free medium, data not shown). Taken together, the ability of KJ-34 to protect against *B. cinerea*, combined with its ability to fix nitrogen, [can increase plant resistance] to pathogens and promote growth. *Burkholderia* bacteria have the ability to degrade environmental pollutants, *B. xenovorans* LB400 can decrease carcinogens (polychlorinated biphenyl, 2-aminophenol, Metallica) (Martínez et al., 2007; Chirino et al., 2013; Reyes-Gallegos et al., 2016). The *Burkholderia* species MBA4 can enrich and bioremediate haloacids from soil (Su et al., 2013). Whether KJ-34 has the ability to decrease environmental pollutants is worthy of exploration.

*Burkholderia* strains produce metabolites that relate to their various functions and eukaryotic hosts (Depoorter et al., 2021; Adaikpoh et al., 2022). The best-known metabolites are bongkrekic acid (BA) and toxoflavin (TF), produced by the food-borne pathogen *B. gladioli* pv. *cocovenenans*, which are highly toxic to humans (Moebius et al., 2012; Gudo et al., 2018). Many metabolites produced by *Burkholderia* strains act as biological control agents, protecting plants against pathogens. *B. cepacia* strains 5.5B and RR 21-2 produce pyrrolnitrin and phenazineto, which inhibit infection with and colonization by *Rhizoctonia solani* (Cartwright et al., 1995; Hwang et al., 2002), and the *B. pyrrocinia* strain Lyc2 synthesizes occidiofungin, which shows antifungal activity against a broad range of plant and animal fungal pathogens (Vial et al., 2007). Other bioactive *Burkholderia* metabolites that have been identified include chitinases, tropolone (Vial et al., 2007), cepacin (Mullins et al., 2019), terpenoids, alkaloids, steroids, anthraquinones, cyclopeptides (Jeong et al., 2003), bactobolins (Seyedsayamdost et al., 2010), enacyloxins, caryoynencin, sinapigladioside, gladiolin and icosalides (Jones et al., 2021), reumycin, enacyloxin, bactobolin (Greenberg et al., 2020), ditropolonyl sulfide, ornibactin, siderophores (Deng et al., 2017), cepabactin, and 2-alkylquinolones (Li et al., 2018; Jones et al., 2021). In this study, the KJ-34-secreted antibiotic metabolites identified by untargeted metabolome included ac-yvad-cho, benzoylstaurosporine, TAXOL C, trichostatin A, thapsigargin, kabiramide B, scopolamine, enniatin B, latrunculin A, rifaximin, rigin, and garcinone C. Ac-YVAD-CHO is a caspase-1 inhibitor that suppresses bacterial infections by mediating ATP release (Xiang et al., 2013). Benzoylstaurosporine (staurosporine analogue CGP 41251), a highly specific inhibitor of protein kinase C, may have the potential for biocontrol (Killion et al., 1995). Trichostatin A, a histone deacetylase inhibitor, is a potential inhibitor against rice blast fungus (Shanmugam et al., 2019). Latrunculin A affects actin filament assembly and relative to plant immunity in wheat against rust fungi (Zhang B. et al., 2017). Garcinone C has antimicrobial activities and induces apoptosis and reactive oxygen species accumulation (Zhang B. et al., 2017; Ibrahim et al., 2019). We carried out semipreparative fractionation of KJ-34 and tested the antimicrobial activity of the predominant metabolites. In conclusion, KJ-34 has a broad antimicrobial spectrum by secreting various known and unknown bioactive compounds. KJ-34 has great potential for use in the development of biocontrol agents and eco-friendly pesticides.

## Data availability statement

The original contributions presented in the study are included in the article/**Supplementary Material**. Further inquiries can be directed to the corresponding author.

## Author contributions

CY, ZW, TQ, JW, and LZ: investigation and validation; CY and ZW: resources; CY, ZW, and TQ: data analysis and manuscript writing; TQ, JW, and LZ: project administration, manuscript writing–review and editing, and funding. All authors contributed to the article and approved the submitted version.
