# Supplementary material for: Burkholderia gladioli strain KJ-34 exhibits broad-spectrum antifungal activity
Source: Front Plant Sci. 2023 Mar 3;14:1097044. doi: 10.3389/fpls.2023.1097044 (PMC10020716; doi:10.3389/fpls.2023.1097044)
Supplement: Supplementary file 2 [file Table_2.docx]

Supplementary Material

# Supplementary Figures and Tables

**
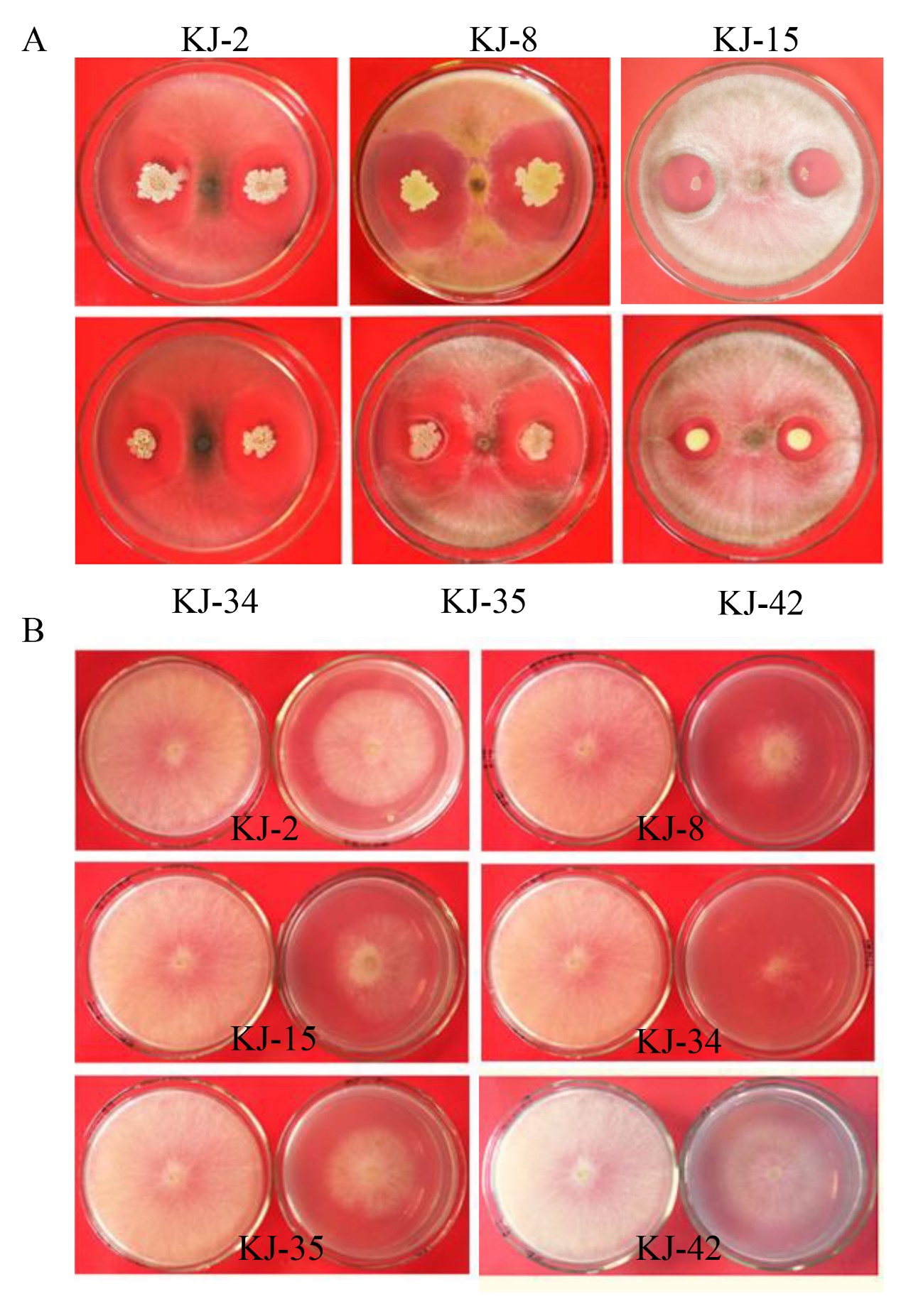
**

Figure S1: The inhibitory effect of isolated bacteria on growth of the pathogens.

**
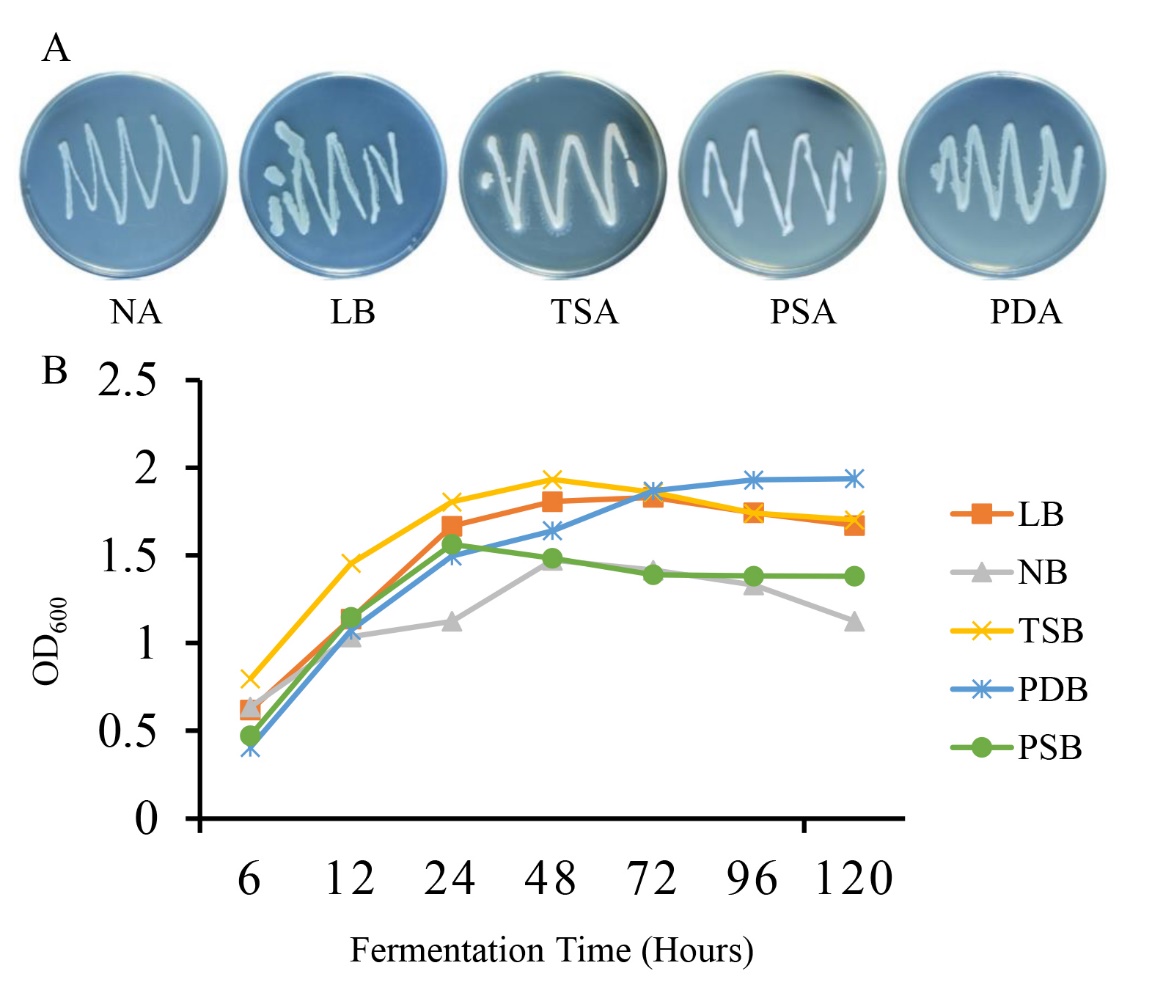
**

Figure S2: Growth profiles of strain KJ-34. (A) Growth velocity and morphology of KJ-34 on different culture medium (LB, PDA, PSA, NA, and TSA). (B) Bacterial concentration (OD_600_) of KJ-34 in liquid medium were measured after inoculated 6, 12, 24, 48, 72, 96, 120 hours.

**
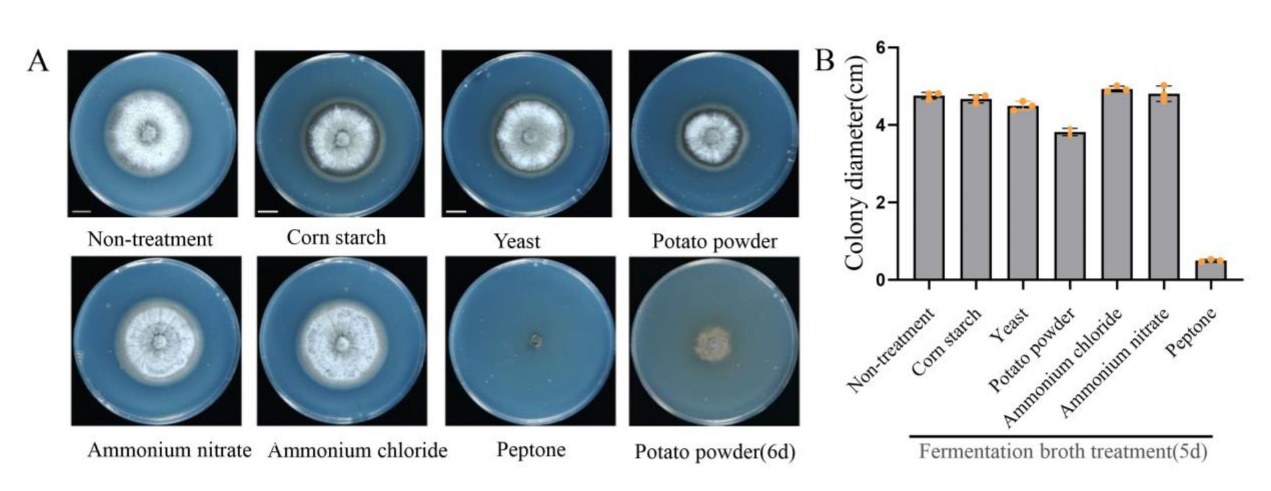
**

Figure S3: Effects of the different carbon and nitrogen component on the ability of KJ-34 antifungal activity. (A) Fermentation filtrate of KJ-34 shake in different medium component after inoculated 5 days were used to test the inhibitory effect of *M. oryzae*. (B) Colony diameter of *M. oryzae* were measured after inoculated 5 or 6 days.

**
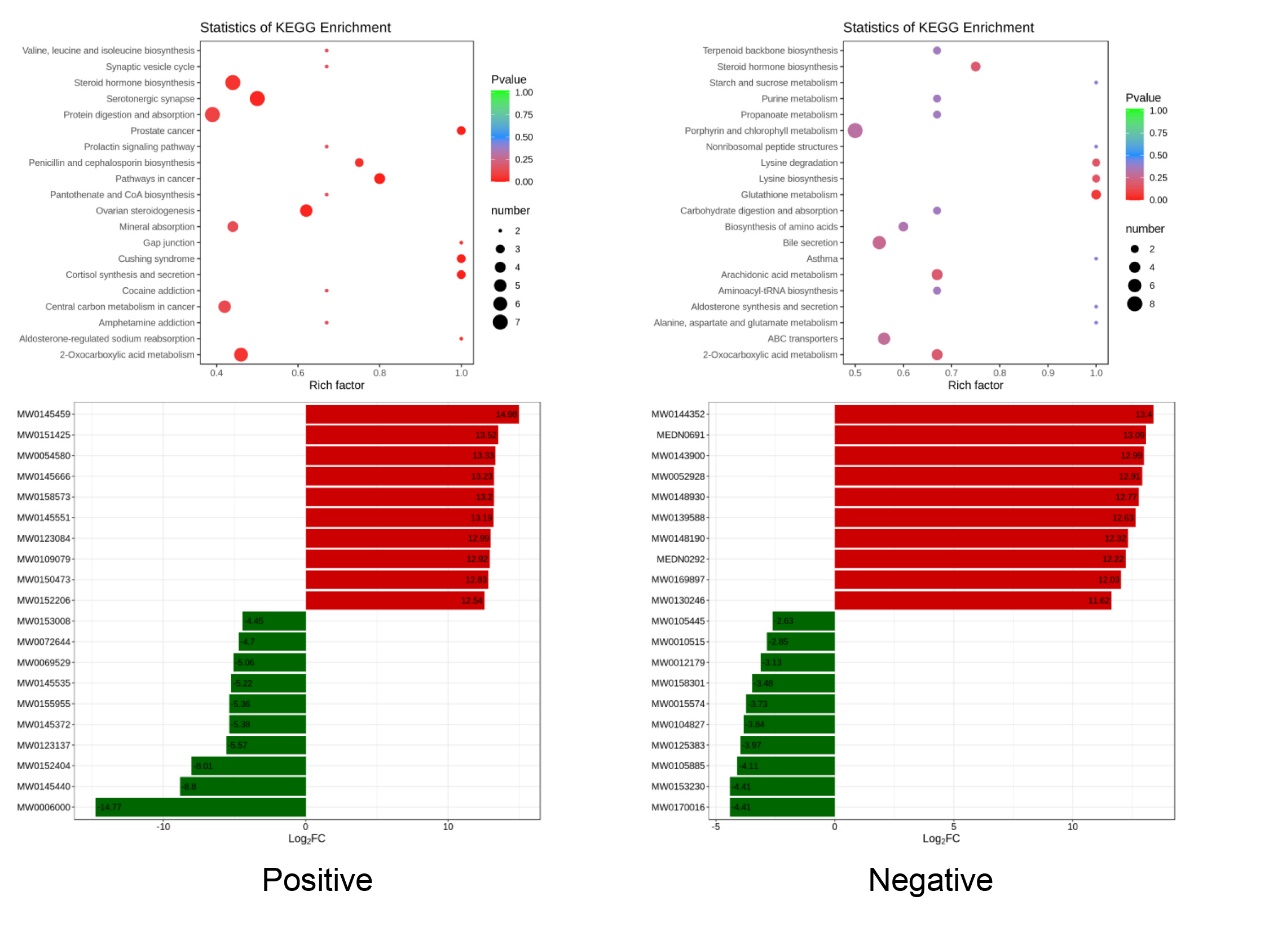
**

Figure S4: Differential metabolites Bar chart. Red represents up-regulation of metabolite content and green represents down-regulation of metabolite content. KEGG enrichment analysis of differential metabolites.

Table S1: The pot experiment of KJ3-4 fermentation filtrate treatment of tomato against *Botrytis cinerea*.

Table S2-S3: Significantly regulated metabolites between groups were list.

Table S4-S5: Differential metabolites were further screen out with the Fold Change greater than 5.
