# Supplementary material for: Burkholderia gladioli strain KJ-34 exhibits broad-spectrum antifungal activity
Source: Front Plant Sci. 2023 Mar 3;14:1097044. doi: 10.3389/fpls.2023.1097044 (PMC10020716; doi:10.3389/fpls.2023.1097044)
Supplement: Supplementary file 3 [file Table_3.docx]

Table S1. The pot experiment of KJ3-4 fermentation filtrate treatment of tomato against *Botrytis cinerea*.

| Treatment | Treatment effect | | Protective effect | |
| --- | --- | --- | --- | --- |
|  | Area of disease | control efficiency | Area of disease | control efficiency |
| 100% Fermentation broth treatment | 2.07±1.62d | 98.20±1.70a | 52.35±10.21d | 75.61±3.39a |
| 10% Fermentation broth treatment | 5.25±9.09d | 96.16±6.65a | 101.30±14.73c | 52.69±5.02b |
| 1% Fermentation broth treatment | 38.24±10.25c | 70.10±6.83d | 116.61±25.32c | 45.81±8.34b |
| Fermentation broth non-treatment | 128.58±21.00a | – | 213.79±13.62a | – |
